# Th2 Cytokines (Interleukin-5 and -9) Polymorphism Affects the Response to Anti-TNF Treatment in Polish Patients with Ankylosing Spondylitis

**DOI:** 10.3390/ijms232113177

**Published:** 2022-10-29

**Authors:** Sylwia Biały, Milena Iwaszko, Jerzy Świerkot, Bartosz Bugaj, Katarzyna Kolossa, Sławomir Jeka, Katarzyna Bogunia-Kubik

**Affiliations:** 1 Laboratory of Clinical Immunogenetics and Pharmacogenetics, Hirszfeld Institute of Immunology and Experimental Therapy, Polish Academy of Sciences, 53-114 Wroclaw, Poland; 2 Department of Rheumatology and Internal Medicine, Wroclaw Medical University, 50-556 Wroclaw, Poland; 3 Department of Rheumatology and Connective Tissue Diseases, Jan Biziel University Hospital No. 2, 85-268 Bydgoszcz, Poland; 4 Ludwik Rydygier Collegium Medicum in Bydgoszcz, Nicolaus Copernicus University in Torun, 85-067 Bydgoszcz, Poland

**Keywords:** interleukin 5, interleukin 9, *IL5*, *IL9*, ankylosing spondylitis, anti-TNF, SNP

## Abstract

Ankylosing spondylitis (AS) is an inflammatory disease that belongs to the spondyloarthritis family. IL-5 and IL-9 belong to the group of Th2 cytokines of anti-inflammatory nature. Polymorphisms in their coding genes have been so far associated with various inflammatory diseases, but there are no reports regarding their involvement in AS pathogenesis to date. The purpose of the study was to investigate relationships between *IL5* and *IL9* genetic variants with AS susceptibility, clinical parameters as well as response to therapy with TNF inhibitors. In total 170 patients receiving anti-TNF therapy and 218 healthy controls were enrolled in the study. The genotyping of *IL5* rs2069812 (*A* > *G*) and *IL9* rs2069885 (*G* > *A*) single nucleotide polymorphisms was performed using the Real-Time PCR method based on LightSNiP kits assays. The present study demonstrated significant relationships between *IL5* rs2069812 and *IL9* rs2069885 polymorphisms and response to anti-TNF therapy. Presence of the *IL5* rs2069812 *A* allele in patients positively correlated with better response to treatment (*p* = 0.022). With regard to *IL9* rs2069885, patients carrying *the A* allele displayed better outcomes in anti-TNF therapy (*p* = 0.046). In addition, *IL5* rs2069812 *A* and *IL9* rs2069885 *A* alleles were associated with lower CRP and VAS values. The obtained results may indicate a significant role for IL-5 and IL-9 in the course of AS and response to anti-TNF therapy.

## 1. Introduction

Ankylosing Spondylitis (AS, synonyms: Betcherew’s disease) is classified as a chronic rheumatic disorder characterised by inflammation of the spine (spondyloarthropathies) but without the presence of rheumatoid factor (RF) which is very often found in other rheumatic diseases. The main symptom of AS is the formation of bone anastomoses between the vertebrae of the spine, resulting in a loss of flexibility. Initially, patients often complain of morning stiffness that clears up after a short period of exercise. Often, the first painful symptoms concern the sacral spine, and as the disease progresses, the pain travels to the higher parts. The lumbar lordosis disappears while the kyphosis in the thoracic spine increases. Inflammation can also affect other joints, e.g., hip, ankle, or knee joints, and tendon attachments, e.g., Achilles tendon attachments. The high degree of disease advancement favours manifestation in other organs, e.g., reduced chest capacity, intestinal ulceration, or uveitis.

AS is more common in men and usually manifests itself around the age of 30. The attendance of patients varies depending on the population, with an estimate of about 0.3–0.5% in the Central European population.

To date, the causes of the development of AS are not fully understood, most likely the pathogenesis includes coexisting genetic, immunological, and environmental aspects (viral infections). A common genetic marker helpful in the diagnosis of AS is the HLA-B27 antigen, but about every tenth patient is negative for this parameter [1].

It is well known that the role of helper lymphocytes, including Th1 and Th2 cells, in the development of autoimmune diseases is significant. Th1 lymphocytes, by secreting pro-inflammatory cytokines, may lead to macrophage-induced inflammation, while Th2-derived cytokines promote B lymphocyte proliferation and humoral responses. On the long arm of human chromosome 5 (murine chromosome 11) there are *loci* for a group of genes encoding Th2 response mediators including such as IL-3, IL-4, IL-13, interferon regulatory factor-1 (IRF-1), and granulocyte-macrophage colony-stimulating factor (GM-CSF) [2]. In our previous work, we discussed the role of Th2 cytokines (IL-4 and IL-13) in inflammatory arthritis [3]. In this study, we focused on single nucleotide polymorphisms (SNPs) in genes coding for IL-5 and IL-9, both of which belong to the mentioned gene cluster on chromosome 5.

First reports on the functionality of IL-5 describe it as a “T-cells replacing factor” (TRF) and a major initiator of B lymphocytes’ differentiation to antibody-producing plasma cells [4]. Now it is well known that human IL-5 has an impact on the process of eosinophil maturation and may trigger eosinophilia. Moreover, IL-5 is involved in allergic reactions by acting on mast cells, causing them to release histamine. Additionally, this cytokine increases the IL-2Rα expression on B lymphocytes and enhances IgA production, as well as triggers the proliferation and differentiation of cytotoxic T lymphocytes (CTLs) [5,6,7].

IL-9 is a factor promoting the pathological expansion of T cells, a mediator of inflammation and has an impact on bone loss [8]. Recently, Kar et al. found that IL-9 is involved in the process of osteoclastogenesis, influencing changes in the expression of genes encoding metabolic active factors, including TNF-α, in patients suffering from rheumatoid arthritis (RA) [9]. Guggino et al. proved that IL-9 has a significant function in the activation of gamma-delta (γδ) T cells in psoriatic arthritis (PsA) patients, which suggests that this molecule is a key but relatively recently recognised player in the pathogenesis of PsA [10].

These observations prompted us to investigate the relationship between the IL5 and IL9 polymorphic variants and predisposition to AS, as well as disease progression in Polish patients with AS. Moreover, we attempted to assess the influence of the studied polymorphisms on the effectiveness of treatment with TNF inhibitors in a group of Polish patients. To our knowledge, the genetic variants of IL5 and IL9 have not been previously studied in AS patients. Both selected polymorphisms have been associated with several immunological diseases [11,12,13,14,15,16,17,18,19,20,21], but they have not been investigated in the context of rheumatic disorders.

## 2. Results

### 2.1. Distribution of IL5 and IL9 Alleles and Genotypes on AS Patients and Controls

Allele and genotype distribution of analysed polymorphisms, *IL5* rs2069812 (located in intron, upstream variant) and *IL9* rs2069885 (amino acid substitution T to M), were in Hardy–Weinberg equilibrium. The *IL5* rs2069812 and *IL9* rs2069885 alleles and genotypes were segregated similarly in AS patients and controls. The *IL5 G* allele and the *IL9 G* allele prevailed in both patients and controls with a frequency of over 70%. However, the *IL9 GG* homozygosity constituted the most frequent of the analysed genotypes that were present in 64% of the patients with AS and almost 69% of the controls (Table 1). There were no significant differences either in the frequency of alleles and genotypes between AS patients and control groups or in the stratified analyses with respect to gender.

### 2.2. IL5 rs2069812 and IL9 rs2069885 Variants and Anti-TNF Treatment Response

**Figure 1:**
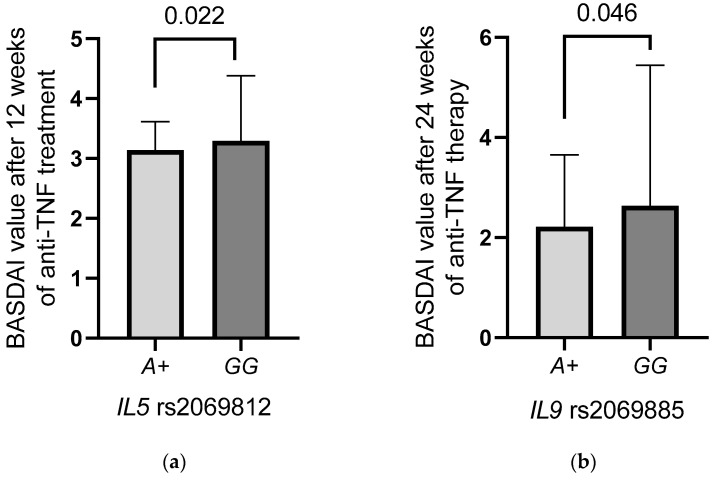
Relationship between the *IL5* rs2069812 (**a**) and *IL9* rs2069885 (**b**) polymorphisms and the BASDAI index in AS patients during anti-TN F treatment.

The group of patients involved in the study underwent treatment with TNF inhibitors with diverse outcomes after 12 and 24 weeks of therapy. Significant relationships between the *IL5* rs2069812 and *IL9* rs2069885 polymorphisms and response to anti-TNF treatment, expressed by the Bath Ankylosing Spondylitis Disease Activity Index (BASDAI) were observed. In both cases, the *A* allele was significantly more frequent in AS patients displaying lower BASDAI values (Figure 1). Carriers of the *IL5* rs2069812 *A* allele showed significantly lower BASDAI values after 12 weeks of anti-TNF therapy than those with the *GG* genotype (*AA* + *AG* vs. *GG* *p* = 0.022, Figure 1a). With regard to the *IL9* rs2069885 polymorphism, the *A* allele was found to be significantly more frequent in patients with a better response to treatment compared to patients with the *GG* genotype (*AA* + *AG* vs. *GG* *p* = 0.046, Figure 1b) after 24 weeks of therapy.

Additionally, heterozygous patients, with the *IL5* rs2069812 *GA* genotype (*GA* vs. *AA* + *GG* *p* = 0.033) and with the *IL9* rs2069885 *GA* genotype (*p* = 0.042) were characterised by lower BASDAI values than those carrying other genotypes, after 12 and 24 weeks of anti-TNF therapy, respectively. These results showed the various impact of selected SNPs on the treatment outcome with a rather earlier effect (at the 12th week) of the *IL5* rs2069812 variants than that of the *IL9* rs2069885 SNP observed after 24 weeks of the treatment.

### 2.3. Clinical Parameters

The frequency of individual alleles and genotypes of the *IL5* rs2069812 and *IL9* rs2069885 genetic variants in AS patients was assessed in relation to selected clinical parameters in AS patients. C-reactive protein (CRP) levels and Visual Analogue Scale (VAS) values were assessed at baseline as well as after 12 and 24 weeks of anti-TNF treatment. The presence of the *A* allele of both *IL5* rs2069812 and *IL9* rs2069885 polymorphisms was significantly associated with lower CRP and VAS values at various time points during therapy.

**Figure 2:**
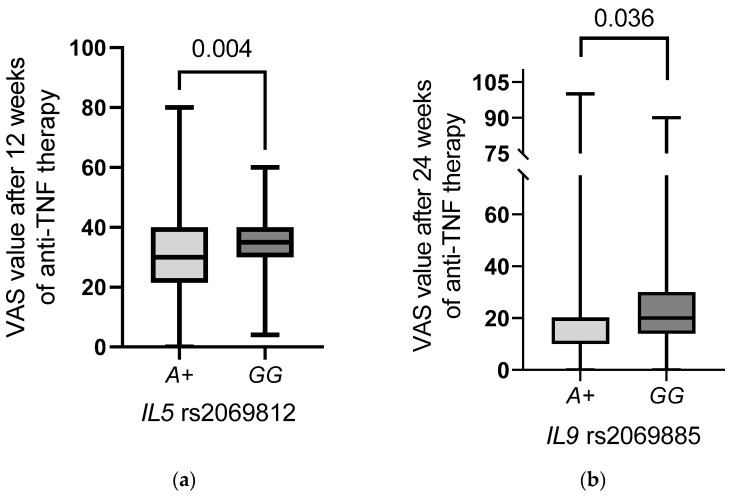
Relationships between the *IL5* rs2069812 (**a**) and *IL9* rs2069885 (**b**) polymorphisms and the VAS values in AS patients during anti-TNF treatment.

The *IL5* rs2069812 *A* allele was detected more frequently in patients with lower VAS values after 12 weeks of therapy than in patients with the *GG* genotype (*GG* vs. *AA* + *AG* *p* = 0.004, Figure 2a).

**Figure 3:**
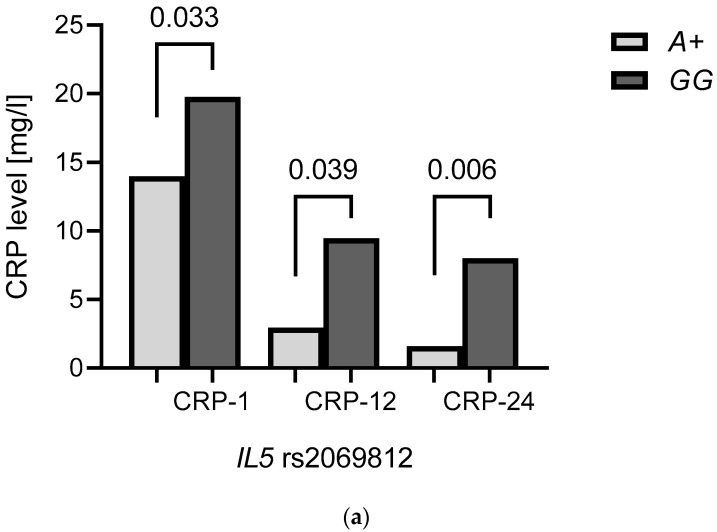

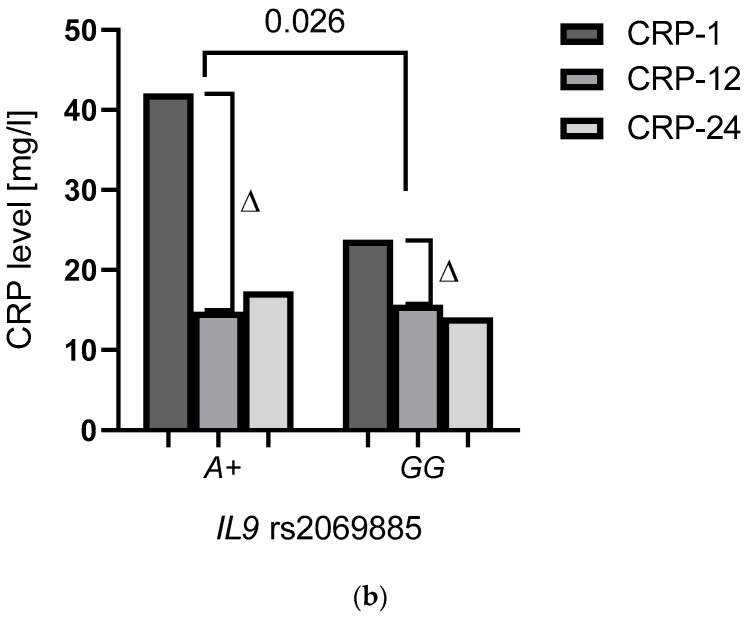
Relationships between the *IL5* rs2069812 (**a**) and *IL9* rs2069885 (**b**) and the CRP values in AS patients during anti-TNF treatment. *A+*—*A* allele carriers (*AG*/*AA* genotypes); *GG*—carriers of *GG* genotypes; CRP-1—at the baseline; CRP-12—after 12 weeks of anti-TNF therapy; CRP-24—after 24 weeks of anti-TNF therapy; Δ—changes in CRP levels after 12 weeks of anti-TNF therapy compared to baseline.

Similar relationships for the *IL5* rs2069812 *A* allele were also observed with regard to CRP levels at different time points of treatment: at the beginning (*p* = 0.033), after 12 weeks (*p* = 0.039), and after 24 weeks (*p* = 0.006) of therapy with TNF inhibitors (Figure 3a). AS patients possessing *IL5* rs2069812 *A* allele displayed decreased CRP levels compared to patients bearing other genotypes.

*IL9* rs2069885 *A* allele was present in patients with lower VAS value after 24 weeks of therapy, compared with *GG* homozygous patients (*p* = 0.036, Figure 2b).

In carriers of this *IL9* rs2069885 *A* allele, a greater decrease in CRP level between 0 and 12 weeks of anti-TNF therapy was observed compared to patients bearing the *GG* genotype (*p* = 0.026, Figure 3b).

Some significant associations were also found for the heterozygous genotypes. The *IL5* rs2069812 heterozygosity was more frequent in patients with lower CRP values after 12 (*GA* vs. *GG* + *AA* *p* = 0.042) and 24 (*GA* vs. *GG* + *AA* *p* = 0.012) weeks of therapy. Similarly, patients carrying the *IL5* rs2069812 *GA* genotype had lower VAS values after 12 weeks of treatment (*GA* vs. *GG* + *AA p* = 0.002). As for the *IL9* rs2069885 *GA* genotype, a greater decrease in CRP levels after 12 weeks (*GA* vs. *GG* + *AA p* = 0.035) and lower VAS value after 24 weeks of therapy (*GA* vs. *GG* + *AA p* = 0.018) were observed.

The above results may suggest that overexpression of heterozygous genotypes may modulate the effect of the presence of individual alleles.

## 3. Discussion

Th2 lymphocytes are mainly responsible for the immune response that protects against parasite infections and hypersensitivity reactions. Numerous literature data report the importance of Th2 cells in the pathogenesis of various autoimmune diseases [22,23,24]. Through the production of cytokines such as IL-4, IL-5, IL-9 and IL-13, Th2 cells participate in humoral immunity, inducing the switching of B lymphocyte classes to IgE production as well as activating eosinophils [25,26,27,28]. The exact molecular mechanism of IL-5 and IL-9 action in the pathogenesis of rheumatic diseases has not been well described to date. The receptor for IL-5 comprises common β receptor subunit, shared with IL-3 and granulocyte/macrophage colony-stimulating factor (GM-CSF), which is essential in signal transduction pathways such as mitogen-activated protein kinase (Ras/MAPK), phosphatidylinositol-3-kinase (PI-3K), Janus Kinase/signal transducers and activators of transcription (JAK/STAT) and Src family kinases (SFKs) [29,30,31,32,33]. In addition, the receptor for IL-9 is also involved in JAK/STAT signalling [34]. Dysregulated activation within these signaling pathways is implicated in the pathogenesis of rheumatic disorders.

The present study was focused on selected polymorphisms in genes encoding IL-5 and IL-9 which belong to the group of Th2 cytokines and have a key role in Th2-mediated immune response [35,36]. Our previous studies documented some associations between polymorphisms in the genes encoding Th2 cytokines and response to anti-TNF therapy as well as clinical parameters of patients diagnosed with AS and other rheumatic diseases such as rheumatoid arthritis (RA) or psoriatic arthritis (PsA) [37,38]. The *IL33* rs16924159 polymorphism was recognised to be significantly associated with disease activity and clinical outcome of anti-TNF agents in both RA and AS patients [37]. In addition, significant associations were found between *IL13* rs20541 polymorphism and response to TNF inhibitors as well as disease severity in RA patients [38].

To the best of our knowledge, genetic variants within genes coding for *IL5* or *IL9* have not been investigated to date, and the present study constitutes the first report in this field. The results of this study suggest the beneficial impact of the presence of the *A* allele in both *IL5* rs2069812 and *IL9* rs2069885 polymorphisms in AS patients receiving anti-TNF treatment. The *A* allele of both studied polymorphisms was found to be negatively associated with clinical parameters such as BASDAI, VAS, and CRP levels in AS patients.

Interestingly, in our previous study involving patients with RA, the presence of the *AA* genotype and *A* allele of the *IL9* rs2069885 polymorphism was significantly associated with lower levels of clinical parameters of inflammation, which might confirm the favourable role of this genetic variant in rheumatic disorders [39].

The *IL5* rs2069812 polymorphism has also been studied in other autoimmune diseases. In two studies comprising patients diagnosed with Graves’ disease (GD), the *G* allele and the homozygous *GG* genotype were more common in patients with GD and were also less frequent in GD patients in remission [14,15]. In a haplotype analysis encompassing other polymorphisms of genes located on chromosome 5, the *G* allele was also significantly associated with an increased risk for GD development. These results are consistent with our report pointing to a favourable role of the *IL5* rs2069812 *A* allele [15].

In addition, similar conclusions regarding the *IL5* rs2069812 polymorphism concern also such diseases as Hashimoto’s thyroiditis or bronchial asthma. In both of these clinical situations, the association between the presence of the *G* allele/*GG* genotype and greater susceptibility to disease development has been reported [11,12]. In adult asthmatics, the presence of the *G* allele was associated with a higher risk of airway hyperactivity development in response to enterotoxins than those with the *AA* genotype [40]. On the other hand, some studies involving patients with non-Hodgkin lymphoma and gastric cancer point to the opposite phenomenon and suggest a protective effect of the *GG* genotype [13,17].

IL-5 has been mainly investigated in asthma and allergic diseases since this cytokine constitutes the most important pathogenic mediator contributing to eosinophilia. High eosinophil levels are a prominent feature in various allergic airway diseases. Immunological studies in IL-5 deficient murine models have demonstrated a diminished eosinophil response upon contact with the parasitic agent [41].

On the other hand, a transboundary line of mice with constitutively expressed IL-5 produced large amounts of eosinophils which confirms the participation of IL-5 in stimulating eosinophil differentiation [42]. Eosinophils are more and more often associated not only with allergic reactions but also with relieving inflammation [43].

It is noteworthy that a regulatory subpopulation of eosinophils (rEos) during the remission of synovial inflammation in RA patients has been described. Moreover, that study revealed that induction of eosinophilic asthma in a mouse model alleviates inflammation and protects the joint tissues. [44]. In addition, another study performed by Chen et al. in two mouse models of inflammatory arthritis reported that parasite-induced Th2 pathway activation leads to an influx of eosinophils to the joint and subsequently relief of inflammation [24]. Additionally, another study demonstrated that the administration of eosinophils in a collagen-induced arthritis mouse model alleviated inflammation [45].

The aforementioned studies strongly suggest that IL-5 might be involved in the pathogenesis of autoimmune and autoinflammatory diseases including rheumatic disorders. For this reason, it is worth exploring the area related to IL-5, as the results of these studies may be useful in developing new therapeutic pathways.

With regard to the rs2069885 polymorphism in the gene encoding IL-9, it was also studied in inflammatory and neoplastic diseases. The team of Sordillo et al. showed that the presence of the *G* allele is related to a higher risk of severe asthma exacerbations in children [20]. In addition, in a haplotype analysis performed by Fatahi et al., the *IL9* rs2069885 *G* allele was associated with a higher probability of developing allergic rhinitis [19]. Interesting results were also obtained in the study by Schuurhof et al. encompassing children with respiratory syncytial virus (RSV) bronchiolitis. The presence of the *IL9* rs2069885 *G* allele was related to a higher risk of severe RSV infection in boys; however, the opposite effect was observed in girls. These results suggest a gender-specific relationship between *IL9* rs2069885 polymorphism and RSV infection in children [46].

The rs2069885 polymorphism was also examined in an extended haplotype analysis of the *IL9* gene in patients with laryngeal squamous cell carcinoma (LSCC). The haplotype containing the *IL9* rs2069885 *G* variant was associated with a lower probability of LSCC development [47]. In addition, *IL9* haplotype analysis also revealed an association of the rs2069885 polymorphism with a predisposition to age-related macular degeneration (AMD) [48].

Despite the lack of reports on the *IL9* rs2069885 polymorphism in rheumatic diseases, there is a number of studies highlighting the role of IL-9 itself in rheumatic diseases. Noteworthy results were obtained in the study performed in 2017 by Rauber et al. in the antigen-induced arthritis (AIA) mouse model. Authors observed that IL-9 deficient mice developed chronic inflammation leading to the destruction of articular cartilage. This phenomenon has been linked to disturbances in regulatory T-cell (Treg) activation. On the other hand, IL-9 administration in mice using a gene therapy approach was associated with the resolution of inflammation and disease progression [49]. However, contradictory results were obtained in a study performed by Gouyou et al. [50]. In this study a therapeutic benefit was not observed in arthritis mice after delivering IL-9-based fusion proteins.

Some of the performed studies regarding IL-9 role in the pathogenesis of rheumatic diseases suggest that this cytokine might contribute to their development. Kar et al. reported that IL-9 modulates the expression of matrix metalloproteinase (MMP) genes and promotes osteoclast differentiation leading to bone destruction in RA [9,51]. In addition, an increase in IL-9 concentrations in the synovial fluid and serum of RA patients has been demonstrated in a few studies [52,53,54]. The pathogenic role of IL-9 in RA development was also suggested in a study by Chowdhury et al. [51]. The authors documented the accumulation of Th9 cells in synovial fluid obtained from RA patients. Moreover, Th9 cells increased neutrophils’ survival and function, favoured differentiation of Th17 cells and enhanced synovial T cell activity and the production of matrix metalloproteinases. In addition, the frequency of Th9 cells in the synovium positively correlated with the activity of RA [51]. Taking into account the aforementioned studies, a conclusion can be drawn about the validity of the use of IL-9 features in the therapy of rheumatic diseases. One of the innovative approaches to stop the degenerative process is mesenchymal stem cell therapy, which has demonstrated the ability to repair cartilage and joints. A study by Sahar et al. reported that this therapy lead to the regulation of IL-9 levels in adjuvant-induced arthritis (AIA), and limited the development of inflammation [55].

In conclusion, the present study is the first to investigate relationships between *IL5* and *IL9* polymorphisms and susceptibility to AS development as well as the clinical outcome of treatment with TNF inhibitors. The obtained results suggest that the *IL5* rs2069812 and *IL9* rs2069885 genetic variants may affect response to therapy with TNF inhibitors in Polish AS patients. The presence of the *A* allele of both *IL5* rs2069812 and *IL9* rs2069885 polymorphisms among AS patients predisposed them to a favorable clinical outcome in anti-TNF treatment. These results imply that *IL5* and *IL9* polymorphisms might be involved in the genetic background of AS. However, the results presented in this study ought to be verified in a study involving a more abundant cohort of patients diagnosed with AS.

## 4. Materials and Methods

### 4.1. Patients and Controls

The study was conducted on blood samples collected from 159 AS patients. Patients were treated at the Department of Rheumatology and Internal Medicine, Wroclaw Medical University, Poland as well as at the Department of Rheumatology and Connective Tissue Diseases, Jan Biziel University Hospital No. 2, Bydgoszcz, Poland. Patients enrolled in the study have been diagnosed with severe AS according to guidelines recommended by the Assessments in Ankylosing Spondylitis International Society (ASAS) and European League Against Rheumatism (EULAR) from 2010. 

In addition, the eligibility criteria were: age over 18 years, Caucasian descent, resistance to treatment with at least two different non-steroid anti-inflammatory drugs (NSAIDs), high disease activity (based on BASDAI values > 4) at the time of qualification and initiation of treatment with TNF inhibitors during the study. Moreover, all patients willing to participate in the study became acquainted with the description of the planned experiments and had to give written informed consent to participate.

The exclusion criteria were as follows: age under 18, ethnic origin other than Caucasian, other autoimmune diseases malignancy, viral infections, pregnancy and breastfeeding as well as lack of willingness to cooperate.

Personal data and clinical parameters collected from patients comprised gender, age, Body Mass Index (BMI), age of disease onset, disease duration, presence of RF and anti-cyclic citrullinated peptide autoantibodies (CCP), Disease Activity Score (DAS-28), BASDAI and VAS. Clinical data were collected at baseline and at 12 and 24 weeks after the initiation of anti-TNF treatment. Detailed characteristics of the study group are presented in Table 2.

The control group included 218 healthy volunteers (90 female and 128 men) with a negative medical history of autoimmune disorders. Moreover, they are honorary blood donors in the Regional Centre of Transfusion Medicine and Blood Bank in Wroclaw.

### 4.2. Single Nucleotide Polymorphisms Selection and Genotyping Methodology

*IL5* rs2069812 and *IL9* rs2069885 polymorphisms were selected on the basis of literature data and analysis of information from databases: PubMed, dbSNP NCBI, SNPinfo Web Server, HapMap, and 1000 Genomes Project. Both of the selected polymorphic variants were associated with the risk of developing diseases autoimmune and neoplastic diseases. The minor allele frequency (MAF) of selected polymorphisms was not less than 10% (1000 Genomes Project). *IL5* rs2069812 (*A* > *G*) is an intron variant and *IL9* rs2069885 (*G* > *A*) is a missense variant in exon 5 of the chromosome.

Peripheral blood from AS patients was collected in EDTA tubes (BD Vacutainer^®^ Blood Collection Tubes). DNA was isolated using the QIAamp DNA Blood Midi/Maxi Kit according to the manufacturer’s instructions (Qiagen, Hilden, Germany). For the determination of polymorphic variants in genes encoding IL-5 and IL-9, the Real-Time polymerase chain reaction (PCR) method was employed based on commercial LightSNiP kits assays (TIB MOLBIOL, Berlin, Germany). Genotyping was performed on a LightCycler 480 Real-Time PCR Instrument (Roche Diagnostics, Basel, Switzerland).

### 4.3. Statistical Analysis

The frequencies of alleles and genotypes of selected polymorphisms in the study and control groups were tested for the Hardy–Weinberg equilibrium. Quantitative clinical values were tested for normal distribution using the Shapiro–Wilk test followed by the Mann–Whitney test for nonparametric data or a *t*-test for normally distributed data. For categorical data, Fisher’s exact test with a two-tailed *p*-value was used. The level of statistical significance was considered to be *p* < 0.05. All statistical analyses were performed using GraphPad Prism 8 software (GraphPad, San Diego, CA, USA).

## 5. Conclusions

In conclusion, the present study is the first to investigate relationships between *IL5* and *IL9* polymorphisms and predisposition to AS as well as clinical outcomes of anti-TNF therapy. The obtained results suggest that the *IL5* rs2069812 and *IL9* rs2069885 genetic variants may affect response to therapy with TNF inhibitors in Polish AS patients. The presence of the *A* allele of both *IL5* rs2069812 and *IL9* rs2069885 polymorphisms among AS patients predisposed them to a favorable clinical outcome in anti-TNF treatment. These results imply that *IL5* and *IL9* polymorphisms might be involved in the genetic background of AS. However, the results demonstrated in this study require validation in larger cohorts of patients diagnosed with AS.

## Figures and Tables

**Table 1:** Distribution of the *IL5* rs2069812 or for *IL9* rs2069885 alleles and genotypes in AS patients.

	AS Patients *N* = 170		Controls *N* = 218	
Allele/Genotype	*IL5* rs2069812	*IL9* rs2069885	*IL5* rs2069812	*IL9* rs2069885
*AA*	12 [7.05%]	6 [3.53%]	14 [6.42%]	6 [2.79%]
*GA*	75 [44.12%]	55 [32.35%]	95 [43.58%]	61 [28.37%]
*GG*	83 [48.82%]	109 [64.12%]	109 [50.00%]	148 [68.84%]
*A*	99 [29.12%]	67 [19.70%]	123 [28.21%]	71 [16.98%]
*G*	241 [70.88%]	273 [80.29%]	313 [71.79%]	357 [83.02%]

**Table 2:** Characteristic of patients with AS.

AS Patients	N = 159
Gender, female [n, (%)]	43 (27.04%)
Age [years]	43.69 ± 13.32
Body Mass Index [kg/m^2^]	25.29 ± 4.28
Disease onset [years]	31.64 ± 9.59
Disease duration [years]	11.75 ± 10.67
HLA-B27+ [%]	~90%
BASDAI (1)	7.55 ± 1.38
BASDAI (2)	3.20 ± 1.64
BASDAI (3)	2.49 ± 2.42
CRP (1) [mg/l]	29.36 ± 55.70
CRP (2) [mg/l]	15.51 ± 31.60
CRP (3) [mg/l]	12.30 ± 26.20
VAS (1) [mm]	80.69 ± 10.75
VAS (2) [mm]	31.48 ± 11.70
VAS (3) [mm]	21.76 ± 16.26
Treatment	
Entracept	29.56%
Adalimumab	42.77%
Infliximab	1.26%
Certolizumab pegol	17.61%
Golimumab	8.81%
MTX	29.59%

(1) at baseline, (2) after 3 months of the anti-TNF treatment induction, (3) after 6 months of the anti-TNF treatment induction.

## Data Availability

The datasets generated and/or analysed during the current study are available from the corresponding author on reasonable request.

## References

[1] Zhang T., Yang F., Zuo K., Wang J., Cheng Z., Zhang J. (2020). HLA-B27 Negativity Is Associated With Renal Function Decline in Patients With Ankylosing Spondylitis and Secondary IgA Nephropathy. Front. Med..

[2] Frazer K.A., Ueda Y., Zhu Y., Gifford V.R., Garofalo M.R., Mohandas N., Martin C.H., Palazzolo M.J., Cheng J.F., Rubin E.M. (1997). Computational and biological analysis of 680 kb of DNA sequence from the human 5q31 cytokine gene cluster region. Genome Res..

[3] Iwaszko M., Biały S., Bogunia-Kubik K. (2021). Significance of Interleukin (IL)-4 and IL-13 in Inflammatory Arthritis. Cells.

[4] Schimpl A., Wecker E. (1972). Replacement of T-cell function by a T-cell product. Nat. New Biol..

[5] Horikawa K., Takatsu K. (2006). Interleukin-5 regulates genes involved in B-cell terminal maturation. Immunology.

[6] Ramos T. (1989). Interleukin 5 is a differentiation factor for cytotoxic T lymphocytes. Eur. J. Immunol..

[7] Schoenbeck S., McKenzie D.T., Kagnoff M.F. (1989). Interleukin 5 is a differentiation factor for IgA B cells. Eur. J. Immunol..

[8] Wu X. (2020). Innate Lymphocytes in Inflammatory Arthritis. Front. Immunol..

[9] Kar S., Gupta R., Malhotra R., Sharma V., Farooque K., Kumar V., Chakraborty S., Mitra D.K. (2021). Interleukin-9 Facilitates Osteoclastogenesis in Rheumatoid Arthritis. Int. J. Mol. Sci..

[10] Guggino G., Ciccia F., Di Liberto D., Lo Pizzo M., Ruscitti P., Cipriani P., Ferrante A., Sireci G., Dieli F., Fourniè J.J. (2016). Interleukin (IL)-9/IL-9R axis drives γδ T cells activation in psoriatic arthritis patients. Clin. Exp. Immunol..

[11] Mestiri S., Zaaber I., Inoubli O., Abid N., Omrani A., Nejehi H., Marmouch H. (2020). Association of cytokine Th2 gene polymorphisms with autoimmune thyroid diseases in Tunisian population. Int. J. Immunogenet..

[12] Polonikov A.V., Ivanov V.P., Bogomazov A.D., Freidin M.B., Illig T., Solodilova M.A. (2014). Antioxidant defense enzyme genes and asthma susceptibility: Gender-specific effects and heterogeneity in gene-gene interactions between pathogenetic variants of the disease. BioMed Res. Int..

[13] Chen Y., Zheng T., Lan Q., Foss F., Kim C., Chen X., Dai M., Li Y., Holford T., Leaderer B. (2011). Cytokine polymorphisms in Th1/Th2 pathway genes, body mass index, and risk of non-Hodgkin lymphoma. Blood.

[14] Inoue N., Watanabe M., Morita M., Tatusmi K., Hidaka Y., Akamizu T., Iwatani Y. (2011). Association of functional polymorphisms in promoter regions of IL5, IL6 and IL13 genes with development and prognosis of autoimmune thyroid diseases. Clin. Exp. Immunol..

[15] Zhu W., Liu N., Zhao Y., Jia H., Cui B., Ning G. (2010). Association analysis of polymorphisms in IL-3, IL-4, IL-5, IL-9, and IL-13 with Graves’ disease. J. Endocrinol. Investig..

[16] Yamamoto N., Sugiura H., Tanaka K., Uehara M. (2003). Heterogeneity of interleukin 5 genetic background in atopic dermatitis patients: Significant difference between those with blood eosinophilia and normal eosinophil levels. J. Dermatol. Sci..

[17] Mahajan R., El-Omar E.M., Lissowska J., Grillo P., Rabkin C.S., Baccarelli A., Yeager M., Sobin L.H., Zatonski W., Channock S.J. (2008). Genetic variants in T helper cell type 1, 2 and 3 pathways and gastric cancer risk in a Polish population. Jpn. J. Clin. Oncol..

[18] Sobota R.S., Stein C.M., Kodaman N., Maro I., Wieland-Alter W., Igo R.P., Magohe A., Malone L.L., Chervenak K., Hall N.B. (2017). A chromosome 5q31.1 locus associates with tuberculin skin test reactivity in HIV-positive individuals from tuberculosis hyper-endemic regions in east Africa. PLoS Genet..

[19] Fatahi F., Chaleshtori A., Samani K.G., Mousavi S.M., Zandi F., Heydari S., Hashemzadeh Chaleshtori M., Amiri M., Khazraee H. (2016). Assessment of the Effects of IL9, IL9R, IL17A, and IL17F Gene Polymorphisms on Women with Allergic Rhinitis in Shahrekord, Iran. Ann. Med. Health Sci. Res..

[20] Sordillo J.E., Kelly R., Bunyavanich S., McGeachie M., Qiu W., Croteau-Chonka D.C., Soto-Quiros M., Avila L., Celedón J.C., Brehm J.M. (2015). Genome-wide expression profiles identify potential targets for gene-environment interactions in asthma severity. J. Allergy Clin. Immunol..

[21] Schürks M., Kurth T., Buring J.E., Zee R.Y. (2009). A candidate gene association study of 77 polymorphisms in migraine. J. Pain.

[22] Jakiela B., Szczeklik W., Plutecka H., Sokolowska B., Mastalerz L., Sanak M., Bazan-Socha S., Szczeklik A., Musial J. (2012). Increased production of IL-5 and dominant Th2-type response in airways of Churg-Strauss syndrome patients. Rheumatology.

[23] Diny N.L., Rose N.R., Čiháková D. (2017). Eosinophils in Autoimmune Diseases. Front. Immunol..

[24] Chen Z., Andreev D., Oeser K., Krljanac B., Hueber A., Kleyer A., Voehringer D., Schett G., Bozec A. (2016). Th2 and eosinophil responses suppress inflammatory arthritis. Nat. Commun..

[25] Gascan H., Gauchat J.F., Roncarolo M.G., Yssel H., Spits H., de Vries J.E. (1991). Human B cell clones can be induced to proliferate and to switch to IgE and IgG4 synthesis by interleukin 4 and a signal provided by activated CD4+ T cell clones. J. Exp. Med..

[26] Punnonen J., Aversa G., Cocks B.G., McKenzie A.N., Menon S., Zurawski G., de Waal Malefyt R., de Vries J.E. (1993). Interleukin 13 induces interleukin 4-independent IgG4 and IgE synthesis and CD23 expression by human B cells. Proc. Natl. Acad. Sci. USA.

[27] Purkerson J.M., Isakson P.C. (1992). Interleukin 5 (IL-5) provides a signal that is required in addition to IL-4 for isotype switching to immunoglobulin (Ig) G1 and IgE. J. Exp. Med..

[28] Carter L.L., Swain S.L. (1998). From naive to memory. Development and regulation of CD4+ T cell responses. Immunol. Res..

[29] Murphy J.M., Young I.G. (2006). IL-3, IL-5, and GM-CSF signaling: Crystal structure of the human beta-common receptor. Vitam. Horm..

[30] Yousefi S., Hoessli D.C., Blaser K., Mills G.B., Simon H.U. (1996). Requirement of Lyn and Syk tyrosine kinases for the prevention of apoptosis by cytokines in human eosinophils. J. Exp. Med..

[31] Ma J., Abram C.L., Hu Y., Lowell C.A. (2019). CARD9 mediates dendritic cell-induced development of Lyn deficiency-associated autoimmune and inflammatory diseases. Sci. Signal..

[32] Ogata N., Kouro T., Yamada A., Koike M., Hanai N., Ishikawa T., Takatsu K. (1998). JAK2 and JAK1 constitutively associate with an interleukin-5 (IL-5) receptor a and bc subunit, respectively, and are activated upon IL-5 stimulation. Blood.

[33] Takaki S., Kanazawa H., Shiiba M., Takatsu K.A. (1994). critical cytoplasmic domain of the interleukin-5 (IL-5) receptor a chain and its function in IL-5-mediated growth signal transduction. Mol. Cell. Biol..

[34] Demoulin J.B., Uyttenhove C., Van Roost E., DeLestré B., Donckers D., Van Snick J., Renauld J.C. (1996). A single tyrosine of the interleukin-9 (IL-9) receptor is required for STAT activation, antiapoptotic activity, and growth regulation by IL-9. Mol. Cell. Biol..

[35] Naora H., Altin J.G., Young I.G. (1994). TCR-dependent and -independent signaling mechanisms differentially regulate lymphokine gene expression in the murine T helper clone D10.G4.1. J. Immunol..

[36] Li Y., Lan F., Yang Y., Xu Y., Chen Y., Qin X., Lv Z., Wang W., Ying S., Zhang L. (2022). The absence of IL-9 reduces allergic airway inflammation by reducing ILC2, Th2 and mast cells in murine model of asthma. BMC Pulm. Med..

[37] Iwaszko M., Wielińska J., Świerkot J., Kolossa K., Sokolik R., Bugaj B., Chaszczewska-Markowska M., Jeka S., Bogunia-Kubik K. (2021). IL-33 Gene Polymorphisms as Potential Biomarkers of Disease Susceptibility and Response to TNF Inhibitors in Rheumatoid Arthritis, Ankylosing Spondylitis, and Psoriatic Arthritis Patients. Front. Immunol..

[38] Biały S., Iwaszko M., Świerkot J., Kolossa K., Jeka S., Bogunia-Kubik K. Association of IL-13 Polymorphism with Response to TNF Inhibitors in Rheumatoid Arthritis Patients. Proceedings of the XVII Congress of the Polish Society of Experimental and Clinical Immunology.

[39] Biały S., Iwaszko M., Świerkot J., Kolossa K., Jeka S., Bogunia-Kubik K. Significance of the Th2 Cytokines Polymorphisms in Rheumatoid Arthritis Anti-TNF Treatment in Polish Population. Proceedings of the 13th International Congress on Autoimmunity.

[40] Losol P., Kim S.H., Hwang E.K., Shin Y.S., Park H.S. (2013). IL-5 Promoter Polymorphism Enhances IgE Responses to Staphylococcal Superantigens in Adult Asthmatics. Allergy Asthma Immunol. Res..

[41] Kopf M., Brombacher F., Hodgkin P.D., Ramsay A.J., Milbourne E.A., Dai W.J., Ovington K.S., Behm C.A., Köhler G., Young I.G. (1996). IL-5-deficient mice have a developmental defect in CD5+ B-1 cells and lack eosinophilia but have normal antibody and cytotoxic T cell responses. Immunity.

[42] Dent L.A., Strath M., Mellor A.L., Sanderson C.J. (1990). Eosinophilia in transgenic mice expressing interleukin 5. J. Exp. Med..

[43] Qin Y., Jin H.Z., Li Y.J., Chen Z. (2021). Emerging Role of Eosinophils in Resolution of Arthritis. Front. Immunol..

[44] Andreev D., Liu M., Kachler K., Llerins Perez M., Kirchner P., Kölle J., Gießl A., Rauber S., Song R., Aust O. (2021). Regulatory eosinophils induce the resolution of experimental arthritis and appear in remission state of human rheumatoid arthritis. Ann. Rheum. Dis..

[45] Liu L., Zhang Y., Zheng X., Jin L., Xiang N., Zhang M., Chen Z. (2019). Eosinophils attenuate arthritis by inducing M2 macrophage polarization via inhibiting the IκB/P38 MAPK signaling pathway. Biochem. Biophys. Res. Commun..

[46] Schuurhof A., Bont L., Siezen C.L., Hodemaekers H., van Houwelingen H.C., Kimman T.G., Hoebee B., Kimpen J.L., Janssen R. (2010). Interleukin-9 polymorphism in infants with respiratory syncytial virus infection: An opposite effect in boys and girls. Pediatr. Pulmonol..

[47] Pasvenskaite A., Liutkeviciene R., Gedvilaite G., Vilkeviciute A., Liutkevicius V., Uloza V. (2021). The Role of IL-9 Polymorphisms and Serum IL-9 Levels in Carcinogenesis and Survival Rate for Laryngeal Squamous Cell Carcinoma. Cells.

[48] Vilkeviciute A., Cebatoriene D., Kriauciuniene L., Zemaitiene R., Liutkeviciene R. (2021). IL-9 and IL-10 Single-Nucleotide Variants and Serum Levels in Age-Related Macular Degeneration in the Caucasian Population. Mediators Inflamm..

[49] Rauber S., Luber M., Weber S., Maul L., Soare A., Wohlfahrt T., Lin N.Y., Dietel K., Bozec A., Herrmann M. (2017). Resolution of inflammation by interleukin-9-producing type 2 innate lymphoid cells. Nat. Med..

[50] Gouyou B., Ongaro T., Cazzamalli S., De Luca R., Kerschenmeyer A., Valet P., Villa A., Neri D., Matasci M. (2021). Antibody-based delivery of interleukin-9 to neovascular structures: Therapeutic evaluation in cancer and arthritis. Exp. Biol. Med..

[51] Chowdhury K., Kumar U., Das S., Chaudhuri J., Kumar P., Kanjilal M., Ghosh P., Sircar G., Basyal R.K., Kanga U. (2018). Synovial IL-9 facilitates neutrophil survival, function and differentiation of Th17 cells in rheumatoid arthritis. Arthr. Res. Ther..

[52] Dantas A.T., Marques C.D., da Rocha Junior L.F., Cavalcanti M.B., Gonçalves S.M., Cardoso P.R., Mariz H., Rego M.J., Duarte A.L., Pitta I. (2015). Increased Serum Interleukin-9 Levels in Rheumatoid Arthritis and Systemic Lupus Erythematosus: Pathogenic Role or Just an Epiphenomenon?. Dis. Mark..

[53] Nowak E.C., Weaver C.T., Turner H., Begum-Haque S., Becher B., Schreiner B., Coyle A.J., Kasper L.H., Noelle R.J. (2009). IL-9 as a mediator of Th17-driven inflammatory disease. J. Exp. Med..

[54] Ciccia F., Guggino G., Ferrante A., Cipriani P., Giacomelli R., Triolo G. (2016). Interleukin-9 and T helper type 9 cells in rheumatic diseases. Clin. Exp. Immunol..

[55] Abd-Elhalem S.S., Haggag N.Z., El-Shinnawy N.A. (2018). Bone marrow mesenchymal stem cells suppress IL-9 in adjuvant-induced arthritis. Autoimmunity.

